# Advancing healthcare through data: the BETTER project's vision for distributed analytics

**DOI:** 10.3389/fmed.2024.1473874

**Published:** 2024-10-02

**Authors:** Matteo Bregonzio, Anna Bernasconi, Pietro Pinoli

**Affiliations:** ^1^Datrix S.p.A., Milan, Italy; ^2^Department of Information, Electronics, and Bioengineering, Politecnico di Milano, Milan, Italy

**Keywords:** data space, distributed analytics, FAIR principles, healthcare, rare diseases

## Abstract

**Introduction:**

Data-driven medicine is essential for enhancing the accessibility and quality of the healthcare system. The availability of data plays a crucial role in achieving this goal.

**Methods:**

We propose implementing a robust data infrastructure of FAIRification and data fusion for clinical, genomic, and imaging data. This will be embedded within the framework of a distributed analytics platform for healthcare data analysis, utilizing the Personal Health Train paradigm.

**Results:**

This infrastructure will ensure the findability, accessibility, interoperability, and reusability of data, metadata, and results among multiple medical centers participating in the BETTER Horizon Europe project. The project focuses on studying rare diseases, such as intellectual disability and inherited retinal dystrophies.

**Conclusion:**

The anticipated impacts will benefit a wide range of healthcare practitioners and potentially influence health policymakers.

## 1 Introduction

In recent years, data-driven medicine has gained increasing importance in terms of diagnosis, treatment, and research due to the exponential growth of healthcare data (1). The linkage of health data from various sources, including genomics, and analysis via innovative approaches based on Artificial Intelligence (AI) advanced the understanding of risk factors, causes, and development of optimal treatment in different disease areas; furthermore, it contributed to the development of a high-quality accessible health care system. However, medical study results often depend on the number of available patient data, crucially when it comes to rare diseases this dependency is accentuated. Typically, the more the data is available for the intended analysis or the scientific hypotheses, the more accurate the results are (1). Nevertheless, the reuse of patient data for medical research is often limited to data sets available at a single medical center. The most imminent reasons why medical data is not heavily shared for research across institutional borders rely on ethical, legal, and privacy aspects and rules. Correctly, data protection regulations prohibit data centralization for analysis purposes because of privacy risks like the accidental disclosure of personal data to third parties.

Therefore, in order to (i) enable health data sharing across national borders, (ii) fully comply with present General Data Protection Regulation (GDPR) privacy guidelines, and (iii) innovate by pushing research beyond the state of the art, this project proposes a robust decentralized infrastructure that will empower researchers, innovators, and healthcare professionals to exploit the full potential of larger sets of multi-source health data via tailored AI tools useful to compare, integrate, and analyze in a secure, cost-effective fashion; with the very final aim of supporting improvement of citizen's health outcomes.

In this paper, we present the Better rEal-world healTh-daTa distributEd analytics Research platform (BETTER), a Horizon Europe Research and Innovation Action that has been conceptualized and designed as an interdisciplinary project consisting of 3 use cases all of which involve 6 medical centers located in the European Union and beyond, where sensitive patient data, including genomics, are made available and analyzed in a GDPR compliant mechanism via a Distributed Analytics (DA) paradigm called the Personal Health Train (PHT) (3).

The main principle of the PHT is that the analytical task is brought to the data provider (medical center) and the data instances remain in their original location. While many classic PHT approaches exist in the literature [see DataSHIELD (4) and WebDISCO (5)], for this project, two mature implementations of the PHT called PADME [Platform for Analytics and Distributed Machine Learning for Enterprises (6)] and Vantage6 [priVAcy preserviNg federaTed leArninG infrastructurE for Secure Insight eXchange (7)] will be fused and adopted as building blocks for the proposed BETTER platform. PADME has been developed by the Klinikum Der Universitaet Zu Koeln (UKK) and has already proven successful in several clinical use cases in Germany. Similarly, Maastricht University (UM) implemented and publicly released Vantage6 (8), a PHT paradigm successfully applied in many real-world healthcare use cases. UM showcased how to perform DA with horizontally (9) and vertically (10) partitioned data in different disease areas, namely oncology (9, 11–15), cardiovascular diseases (10, 16), diabetes type 2 (17), and neurodegenerative diseases. This work shows that federated learning in the healthcare domain is technically feasible, and shows a historical track record and knowledge of applying federated learning in the medical domain while knowing the challenges to scale and adoption, which are addressed in this project.

The clinical use cases we consider focus on evidence-based research on the following pathologies:

(1) Paediatric Intellectual Disability,
(2) Inherited Retinal Dystrophies, and
(3) Autism Spectrum Disorders.

Within those use cases innovative digital tools, technologies, and methods will be researched, developed, and validated in real-world scenarios. In this paper, our focus is on the catalyst role that distributed analytics can have in the field of e-health interventions, contributing to the transformation of the field of health services at an EU-wide level.

## 2 Building the research agenda

During the early design of our Research and Innovation Action, we came up with the need to define specific objectives and relate them to explicit measurable outcomes in order to help the development of the agenda and the workplan of the action. Below we present each of the three main BETTER research agenda constituents.

### 2.1 Overcome cross-border barriers to health data integration, access, FAIRification, and preprocessing

We aim to guide medical centers in collecting patient data following a common schema in order to promote interoperability and re-use of datasets in scope. This includes legal, ethical, and data protection authorizations, data documentation, cataloging, and mapping to well-established and therefore widely understood ontologies. Attention will be devoted to the FAIRification of the datasets used in the project. This means that the FAIR principles (18) (i.e., Findability, Accessibility, Interoperability, and Reusability) will be guaranteed in the results of the project. We will also focus on the integration of external sources such as, but not limited to, public health registries, European Health Data Space [EHDS, (19)], the 1+Million Genomes initiative [1+MG, (20)] and the European Open Science Cloud [EOSC, (21)].

Legal and ethical implications shall need to be duly considered and procedures for data access and re-use will be proposed. As a default preprocessing step data pseudonymization will be performed to mitigate the risk of personal data leak; this will be followed by data quality and integrity assessment. Finally, this objective enables the integration of a BETTER station at each medical center premises, validating the accesses to the relevant local datasets including genomics.

This first aim builds on the matured experience where cross-border health data integration has been demonstrated on a small scale. Novel concepts and approaches will be researched and developed to address BETTER integration of multiple data sources, interoperating with public health data repositories via BETTER, data quality, and integrity assessment algorithm in a distributed fashion. A real-world large-scale data integration framework based on well-established ontologies will be demonstrated accounting for heterogeneous data sources including whole genome sequencing.

### 2.2 Deploy a distributed analytics framework for cross-border data processing and analysis

We plan to deploy, test, and utilize BETTER, a PHT-distributed analytics platform composed of stations hosted at each medical center's premises. Furthermore, a central service will be hosted by UKK in order to monitor and orchestrate activities. Importantly, this framework will support the development of analytics and AI tools via both Federated and Incremental Learning modalities; in line with GDPR data will not leave a single medical center. This framework will be exploited by researchers, data scientists, and software developers to securely build applications for analyzing multiple health datasets including genomics.

Access to cross-border healthcare data is indispensable for innovation; however—currently—it is time-consuming and difficult due to privacy and regulatory concerns (9). Furthermore, to effectively exploit multiple datasets via AI, a common schema and ontology should be applied. Here the ambition regards the deployment of BETTER, a privacy-by-design infrastructure, to all medical centers connecting FAIR data sources and allowing federated data analysis and machine learning. Crucially, patient data never leaves a medical center. To this end, the BETTER platform complements the implementation of EHDS2 (22) as it focuses on the integration of patient data including genomic and other clinical research data thus offering a reference architecture for future synergies between EHDS and 1+MG.

### 2.3 Development of distributed tools leveraging artificial intelligence capabilities

Within each use case, tailored tools are developed in order to properly answer clinical needs. Some of those will indeed exploit DA and AI to push data analysis boundaries going beyond the state of the art. Crucially, multiple data sources including genomics will be fused together aiming to better understand risk factors, causes, and development of the studied diseases. The tools will be developed using a co-creation methodology where medical end-users closely collaborate with researchers and technology providers enabling the emerging new concepts. Finally, trustworthy AI guidelines (23) will be followed throughout the development lifecycle, and particular attention will be devoted to the explainability of the developed tools.

Distributed algorithms iteratively analyze separate databases in order to learn without patient data being centralized (24). Within the healthcare sector, this subject is attracting a lot of attention and enabling important advances (25); furthermore, researchers are actively working on topics such as federated and incremental learning modalities, data and model parallelism, and ensembling techniques (26). This objective aims to research and apply novel computer vision, machine-, deep-, and reinforcement-learning techniques and apply them to health-related real-world data available in the use cases under study.

Apart from the above, there are other important aims that the project supports such as the ELSA (ethical, legal, and societal aspects) awareness in the AI lifecycle and aspects related to the planning, coordination, and implementation of the different medical use cases, on which we do not elaborate as they are not related to the core aspect of distributed analytics as a means to change our approach on real-world data integration.

## 3 The technology constituents

**Figure 1:**
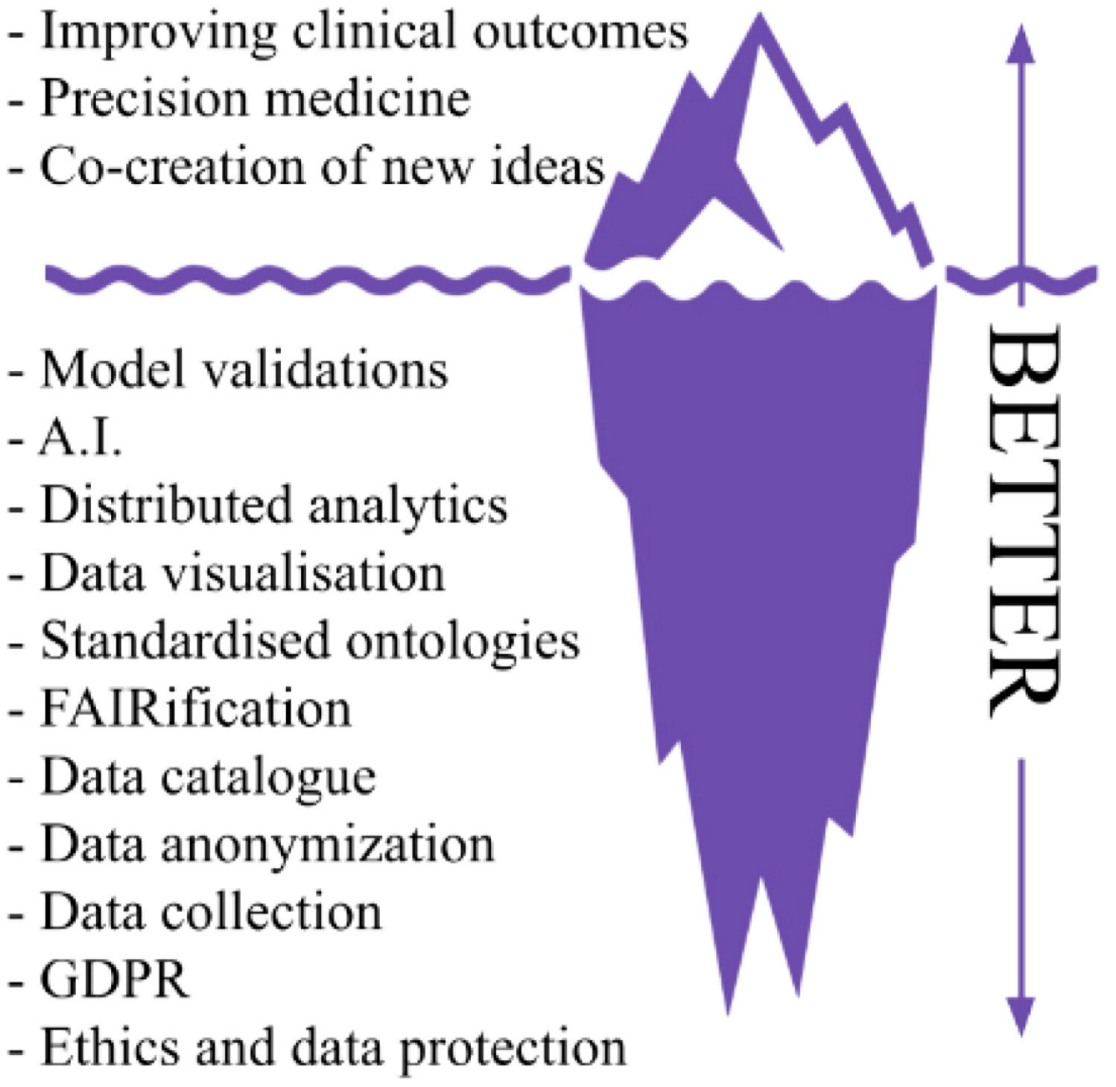
Iceberg representation of needed tasks to achieve precision medicine and innovation in real world scenarios, readapted from Subbiah (2).

The BETTER project builds on the experience gained by UKK on PADME in deploying security-by-design PHT infrastructures in several real-world scenarios enabling medical centers to share and analyze multi-sources health data via a federated learning paradigm in a GDPR compliant mechanism. The importance of this result is also highlighted in a recently published Nature article (2) about the next generation of evidence-based medicine, the authors present an iceberg where evidence-based medicine represents only the tip of the iceberg, while the vast amount of different and heterogeneous data sources and processing tasks represent what lies underneath the surface. The author argues that “a deep synthesis and amalgamation of all available data is needed to achieve next-generation, deep evidence-based medicine”. Figure 1 exploits the iceberg analogy to summarize the BETTER contributions.

In line with the emerging concept of the European Health Data Space, BETTER aspires to offer a lighthouse implementation of healthcare distributed analytics via a multidisciplinary framework based on PADME that supports better healthcare delivery, better research, innovation, and policy-making and, indeed enables medical centers to make full use of the potential offered by a safe and secure exchange, use and reuse of health data. Furthermore, three real-world use cases addressing different medical domains will be demonstrated and specific tools based on the latest technology and AI will be developed to address clinical needs in an innovative way, aiming to achieve results that go beyond the state of the art. BETTER will showcase a consistent, trustworthy and efficient set-up for the use of health data, including genomics, for clinical decision support.

The proposed platform will follow an inclusive, rich inter- and trans-disciplinary methodology, not only across scientific disciplines but also facilitating and promoting knowledge sharing between universities, Small and Medium Enterprises, and healthcare professionals. An Agile methodology will be adopted and a shared ‘language' will be built to effectively close gaps between scientific knowledge, clinical needs, policy changes, and technological issues in a broader sense. Contrary to a waterfall model, ideas, prototypes, and discussions will constantly loop through the project's beneficiaries for early validation and fast development. Vitally, brainstorming, collaborative design, and scientific contamination will be promoted across use cases by actively engaging (calls, meetings, workshops, events, etc.) researchers, technology providers, healthcare professionals, and relevant stakeholders.

### 3.1 The overall BETTER platform

As per the PADME framework, BETTER relies on the concept of "bring-computation-to-data" via incremental and federated learning, which avoids unnecessary data moving across medical centers while exploiting much of the information encoded in such data (1). The intuition behind BETTER can be explained via a railway system analogy which includes *trains, stations*, and train *depots*. The train uses the network to visit different stations to transport, for example, several goods. By adapting this concept to BETTER, we can draw the following similarities:

- The *Train* encapsulates an **analytical task**, which is represented by the good in the analogy.
- The **data provider** takes over the role of a reachable *Station*, which can be accessed by the Train. Further, the Station executes the task, which processes the available data.
- The *Depot* is represented by our **central service** including procedures for Train orchestration, operational logic, business logic, data management, and discovery.

**Figure 2:**
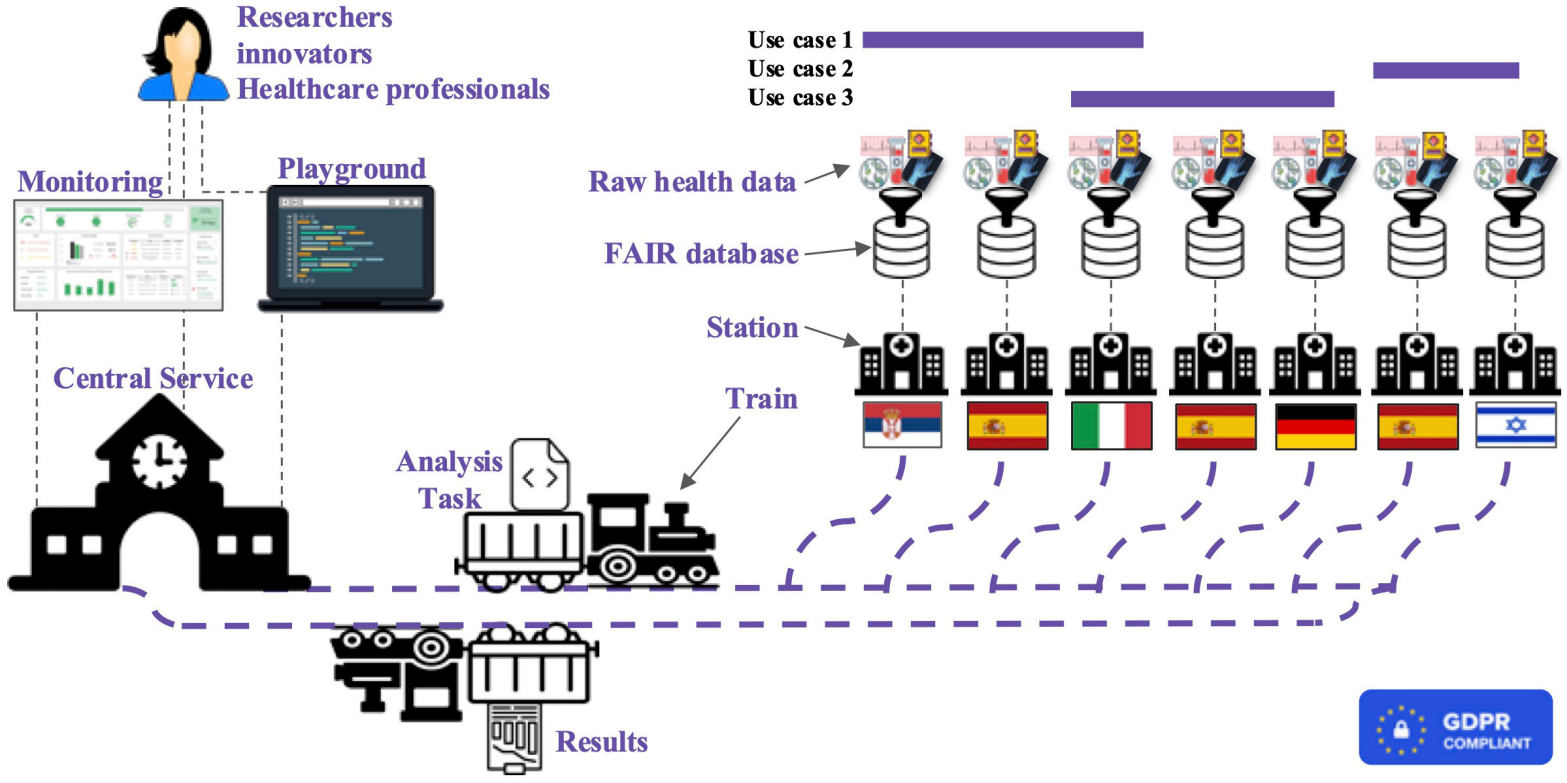
High-level representation of the BETTER platform where researchers, innovators, and healthcare professionals can run analysis and receive results exploring cross-boarders health data.

Thus, from a top-level perspective, the main infrastructure components are Trains, Stations, and Central Service; furthermore, additional modules are available for privacy and security enforcement. An overview of the whole BETTER platform system can be observed in Figure 2; the main constituents are detailed below.

#### 3.1.1 Trains

A Train needs to encapsulate code to perform certain analytical tasks, which are executed at distributed data nodes (the Stations). As it performs its duty, a Train travels from one Station to another and executes commands on-site, utilizing the data available in each location. Thus, the result of the analysis is built incrementally and can be anything, based on the Train code. To achieve this result, the code of the train is encapsulated in an Open Container Initiative-compliant [see The Linux Foundation (27)] image where the code is encapsulated along with all the required dependencies, thus eliminating the need for Train developers to handle the diverse execution node environments. Moreover, in order to increase transparency, a Train stores metadata information about the data the code is accessing, the type and intent of the analysis and its creator. In order to enhance security, a Train must be instantiated exclusively from a Train Class that is stored within an App Store. The App Store is a repository of Train Classes; ahead of being published in the App Store each Train Class is examined by the community and/or by automatic procedures to detect malicious code in particular to prevent disclosure of Stations private data. During their lifecycle, trains can be in several states. First, a Train Class which passed security checks is created and stored in the App Store. When a researcher or an innovator wants to conduct a data study, they select a suitable train from the App Store, and a new instance of the Train is created. Subsequently, the Train moves to an idle state, waiting to be moved to a Station; after the transmission, the Train remains in the idle state at the Station and applies to achieve the permit to be executed. If the Station Administration grants the permission the Train changes its state to running. At this stage, two scenarios may happen:

(a) The Train execution is successful, and the Train is sent back to the Central Service to be routed to the next Station;
(b) The Train execution fails: in this case, the Train is however sent back to the Central Service for code analysis and debugging.

#### 3.1.2 Stations

A Station is a node in the distributed architecture that holds confidential data and executes the code of the trains. In the most common scenario, a Station corresponds to an institution, hospital, or department. Each Station acts as an independent and autonomous unit. Each Station has two main components: (a) the data and (b) the software (i.e., the container executor). Stations receive trains to be executed; however the execution is not automatic by default but rather the Station administration has to grant permission and can reject the Train, for example, due to doubts about the data usage or a lack of capacity. Anyhow, Station administration can also configure that Trains with specific characteristics are automatically executed. When the execution of a Train terminates, the Station administrator checks the results of the Train. The train can be rejected if the results contain confidential data. In addition, every Station offers a visual interface that serves as a control panel for the Station administration to coordinate the Trains' execution cycle. To summarize, each Station has to:

(a) Manage the permission applications for controlling access to the confidential data stored within the institution;
(b) Execute the Trains producing the partial results in the context of incremental use of the federated analytical framework.

### 3.2 The BETTER central service

The Central Service component provides three types of services: (a) a metadata repository to allow data discovery; (b) management tools for Train creation, secure transmission to Stations, orchestration, monitoring, and debugging; and (c) a repository of pre-trained trains that can be directly used by healthcare professionals on their own data to get the results of an AI-based method that has been iteratively trained on data from various institutions. The metadata repository of the Central Service, for each health record datum (e.g., radiology image, genetic test) stores (a) information about its type, the format in which the information is encoded, and the protocol and technologies that have been used for its production; (b) anonymized patient-related data (of particular relevance for patient stratification and longitudinal studies); and (c) information on the location of the datum (i.e., the custodian Station where the datum resides). Notice that only metadata are reported, not the data itself, which is only stored at the corresponding Station. The metadata repository is constantly updated by means of federated queries to all the Stations affiliated with the distributed architecture. The data in the metadata repository are stored using terms from well-known and standard pipelines, in order to maximize the interoperability of data from different institutions. The Central Service also provides all the management tools to allow the creation of a Train by a scientist from a Train class in the App Store, the secure transmission to and from Stations, and the notification to the scientist.

Finally, Trains that have been executed on several Stations and have been analyzed both by each of the Station Administrators and by the involved partners to ensure that they do not disclose protected data, can be stored in a repository of pre-trained Trains. We envision such Trains to perform future complex AI-powered operations (e.g., evaluate the condition of a patient from a medical record); a healthcare professional can clone a pre-trained Train on their local environment and execute it on their local data to get predictions. In this way, both patients and healthcare professionals can benefit from AI-based solutions, without the need to design the analysis and train the model. Moreover, as the execution is local, no private data is disclosed.

A web-based monitoring interface will ensure users with different roles access with respect to different content and functionalities within the BETTER platform. Researchers, innovators, and healthcare professionals will be able to perform analysis through the Central Service, as well as share results with other researchers and professionals to enhance cross-border collaboration in medical investigation. Moreover, policymaker user access will be also available with a high-level reporting view to easily see trends, and patterns and identify unexpected events. This will enable policymakers to identify problems and take data-driven corrective actions.

#### 3.2.1 Monitoring component

The BETTER platform includes a Train metadata schema that provides detailed information about each Train, allowing Train requesters and Station administrators to access relevant data such as Train location and status. Each Station is also equipped with a metadata Processing Unit that collects and stores static metadata about the Station and dynamic Train execution information such as current state and processing unit usage. This dynamic data is converted to conform with the schema standard and transmitted to a global Train metadata repository located in the Central Service. The Station administrators can also apply customizable filters to the metadata stream, allowing them to maintain control over the outgoing processes through a web-based monitoring interface. If PADME is used, a reference to the available metadata schema is in the documentation (28).

#### 3.2.2 Playground component

The research and development of AI-driven analysis on distributed health data today still represents a significant challenge due to the complexity and limited literature available. Based on experience, researchers and developers require time and practice to familiarize themselves with the infrastructure and overcome the initial complexity. To this end, BETTER provides a Playground component that allows exploration, test, and validation of analysis tasks. For instance, it allows for:

(1) Testing criteria including proper connection interface, matching schema, error-free analysis execution, and correct result storage structure.
(2) Validate connection interface: the DA algorithm must be able to connect to the data source properly. This means the configuration of the algorithm should match the data source's connection interface. All connection credentials (such as file path, hostname, port, and type of database) should be correct.
(3) Assess matching schema: the DA algorithm should be able to send correct queries to the data store and receive the corresponding results. Hence, the expected data schema of the analysis task should match the actual schema of the data source.
(4) Error-free execution: if the connection interface and matching schema are correct, the DA algorithm should execute without any errors. This means the program should terminate with exit code 0, indicating a successful program execution.
(5) Correct result storage: the analysis results should be stored in the correct location and format. The code should emit the results as a file or a processable bit string for transmission. An initial implementation of this component is available and documented on PADME [see Weber and Welten (29)].

#### 3.2.3 Privacy and security enforcing components

The privacy and security components can be subdivided into two aspects: (1) components for user authentication and (2) permission management and components for secure transmission and lifecycle handling of Trains.

Regarding the first aspect, the access to the Central Service (that allows to request a train and to query the metadata repository) is controlled by an Identity and Access Management (IAM) component which manages user accounts and access authorization.

Regarding the second aspect, Trains' life cycle handling, as per PADME the architecture follows several design principles to protect sensitive data. One assumption is that the station admin, who is interacting with the Station software, is authorized to inspect and release potentially sensitive data, which has been generated in the context of the Train execution (e.g., a query result or model parameters). However, the admin's authority is limited and is only valid within the institutional borders. Therefore, the admin must not see the results of the preceding stations. The admin further should also be sensitized to the intrinsic activities of the executed Train and the files inside the Train, which will be released after the Train has left the institution. To meet these requirements, the Station software incorporates a mechanism to inspect the Train contents and visualize added, changed, or deleted files. In addition, in case the Train produces query results, the admin is able to audit the file contents themselves. The software detects the changes and only visualizes data, which is relevant to the current station by simultaneously hiding information from other stations.

For transmission, BETTER adopts a private-public key encryption policy; the assumption here is that the Central Service is considered trusted. The accomplishment of a secure transmission is made possible through the implementation of an encryption process that ensures no Train is stored in an unencrypted form and only the intended recipient has the ability to decrypt it. This strategy specifically employs the utilization of private and public keys for each involved entity, including the Train requester, Central Service, and Stations. First, the train requester instantiates a Train instance, which is encrypted by a symmetric key. This symmetric key is generated for each Train request ad hoc. In the second step, the symmetric key is encrypted by the public key of the first station. After the Train transmission, the Station reversely decrypts the Train, executes it, and re-encrypts it with the public key of the Central Service. This procedure is repeated for each Station in the route. At the end, the final Train including the encrypted aggregated results is stored in the Central Service encrypted such that only the requester is able to inspect the results.

### 3.3 Deployment at each medical center

The proposed platform requires that a dedicated hardware (server) is deployed within each medical center premises; this server actually implements the Station and ensures the availability of computational power. As per PADME, the integration of a new Station (medical center) in the BETTER ecosystem is done through an "Onboarding Service." The service includes registration to the BETTER Station Registry and setting up the Station Software. The Station Registry is a central service that allows users to onboard, register, and manage station information. It provides an overview of stations. A new station is registered by filling in the station data. The Station Registry also generates public/private keys and the .env file for each onboarded station. The Station Software can be configured by following the provided web browser-based wizard steps. Station Software is a local software component that is installed on the medical center site. Station software provides a graphical user interface as a management console to coordinate the Train execution cycle. The connection from Station Software to the medical data source that is kept in the medical center server should be configured by the Medical center IT department. As a reference, well-documented deployment instructions are available on PADME documentation (30).

## 4 Data aspects

Better integration and use of health-related real-world and research data, including genomics, for improved clinical outcomes, is gaining more importance in the last years. The context to this relates to the fact that researchers, healthcare professionals but also science entrepreneurs shall benefit from better linkage of health data from various sources, including genomics, based on harmonized approaches related to data structure, format, and quality. This shall be further useful as they will have access to advanced digital tools for the integration, management, and analysis of various health data re-used in a secure, cost-effective, and clinically meaningful way enabling the improvement of health outcomes. Below we present our approach toward data FAIRification and data fusion in the BETTER project.

### 4.1 Data FAIRification

#### 4.1.1 Rationale

A solid infrastructure that is able to organize and share the needed information at the central level (thus enabling also pair-wise interchanges between the data providers' stations) is needed. As a fundamental approach for designing the metadata, the BETTER project will follow the DAMS proposal (1), which has introduced a foundational metadata schema to allow DA infrastructures to comply with FAIR principles (18). The DAMS schema comprises two categories of metadata: those related to Trains and those related to Stations. Trains must be described by (1) *Business information* (e.g., the author of the Train algorithm); (2) *Technical information* (e.g., the data type the algorithm is processing); and (3) *Dynamic execution information* (e.g., the log output the Train is producing). In parallel, Stations must contain (1) *Business information* (e.g., the location of the data provider); (2) *Runtime environment information* (e.g., capabilities in terms of computational power); (3) *Data information* (e.g., size of a dataset or the data type provided by the station).

The choice of which attributes including within each entity dimension of the repository will be crucial for fulfilling the FAIR data principles requirements. In line with the DAMS approach, we plan on aligning our business information to the DataCite Metadata Schema (31), which assigns digital object identifiers to both trains and station assets and ensures that sufficient information is available for each of their components. The Friend of a Friend ontology (32) can be employed to express business information about social entities (such as the owners of trains and stations). The technical information of the train and the data information of the data provider will be aligned with the Software Ontology (33). The Data Catalog Vocabulary may be used to provide predefined attributes describing the semantics of data sets (34).

Differently, clinical data types and related metadata are typically specific to the context of use, leveraging the characteristics of the disease, of patients, and relevant parameters for the problem at hand. BETTER is prepared to address the data management problem with a general approach. As these data types are not covered in DAMS, their management will be inspired by extensive previous work in the field [conducted within the "Data-driven Genomic Computing" ERC AdG n. 693174 (35)]. More specifically, four directions in the agenda of BETTER will be followed to guarantee the scalability of semantic/syntactic standards of clinical data types:

1. We will ensure interoperability at the level of the same pathology by having the partners generate datasets that agree upon the same standards.
2. We will employ a data schema that captures the main properties of a generic clinical context, keeping a high abstraction level to encourage maximum interoperability [examples are the Genomic Conceptual Model (36) and the COVID-19 Host Genetics Initiative Data Dictionary (37)]. Typically, clinical data involve demographic (or static) information on the patient and longitudinal measurements related to medical encounters, treatment, or laboratory measurements.
3. We will use a key-value paradigm for information that is not shared among different pathologies and that is specific to a given use case, thus creating a very flexible and expressive data model that allows storing all relevant information without dealing with integration and interoperability at the storage level [see Masseroli et al. (38)].
4. We will perform semantic annotation by using, predominantly well-adopted terminologies such as NCIT (39), the International Statistical Classification of Diseases and Related Health Problems (ICD) at its most updated version [11th revision, (40)], and Logical Observation Identifiers Names and Codes [LOINC, (41)]. For other information, we will employ dedicated biomedical ontologies as we described in Bernasconi et al. (42), sourcing them from BioPortal (43) and Ontology Lookup Service (44). In this way, we will pursue complete semantic interoperability between the metadata associated with known ontology.

For genomic data, the BETTER project will initially acquire DNA and RNA sequencing data in both FASTQ and BAM formats. All submitted sequence data will be aligned using the latest human reference genome; variant and mutation calls will output VCF and MAF formats, whereas gene and miRNA expression quantification data will be kept in TSV format. Other genomic signals for tertiary data analysis will be homogenized according to guidelines of the Global Alliance for Genomics and Health (45).

#### 4.1.2 Approach

As a first step, BETTER deals with datasets discovery at each medical center. Multiple focus groups will be organized with both technical and clinical stakeholders to understand in depth the available datasets, more specifically: (1) dataset characteristics and size (to support *findability*); (2) data types with their attributes and value ranges (useful to *interoperability and reusability*); (3) pathology-related interpretation (to assess *interoperability* aspects); (4) examples of data usage in real-world scenarios (to foster *reusability*). Dataset profiling activities will be conducted manually and with available tools (46). They will allow to measure the overall value of the data at hand, assessing typical data quality metrics such as coherence, completeness, as well as the heterogeneity of the attributes, which are possible feature candidates. For what regards genomic data, we will evaluate the possibility of re-running bioinformatic pipelines to homogenize the collected data among different centers.

Secondly, the project tackles datasets' pseudonymization. By default, data will be pseudonymized before joining the BETTER platform, which requires the implementation of modules for: (1) identifying personal data from images and text; (2) pseudonymization of reference ID to preserve leakage between same patient samples; (3) where applicable, defacing of face images.

Thirdly, we will develop a unified schema repository for medical centers' data and metadata integration. A unifying global model will be designed to accommodate all the data formats and their describing metadata, and serve as a reference for the next analysis steps.

Finally, we will deal with FAIRification of medical centers' datasets. We will research and develop dedicated preprocessing and ETL (Extract, Transform, and Load) processes to onboard health datasets from each medical center to BETTER (allowing the *accessibility* principle within the PHT framework); this task will achieve data FAIRification by scheduling transformation functions to adjust the initial content into appropriate destination formats (making it *findable* through appropriate metadata). Medical-center-specific data formats, protocols, and characteristics will be mapped to a standard schema, enabling *interoperability* and, eventually, distributed analytics–available for future *reuse* in other European-level infrastructures. Building on previous research of the projects' participants, user-friendly FAIRification instruments will be preferred, and re-using and enhancing existing open-source packages, such as University M (47), will be encouraged.

To achieve a completely interoperable format of the metadata repositories, we will provide their content in standardized formats such as Resource Description Format (RDF) or JavaScript Object Notation (JSON). These standards make it unnecessary to know the internal structural organization of a specific data provider in order to successfully execute a Station data retrieval query. Moreover, RDF/JSON data store approaches are sufficiently flexible to describe arbitrarily complex concepts without the need to redesign the providers' databases. Eventually, we will allow the metadata information to be queried through APIs internal to the project participants. Genomic, image, and clinical data, instead, as they will not be shared, will have to comply with specific data formats that will be indicated in the metadata schema. The proposed ecosystem will be designed so that it complies with any main data storage technology and, therefore, also with emerging standards like the Fast Healthcare Interoperability Resources (FHIR) (48).

#### 4.1.3 State of the art

Implementing FAIR principles in the context of a big distributed platform is an effort that concerns multiple aspects. While the literature offers many contributions in the areas of FAIR principles interpretation (49, 50) FAIRness assessment (51–53), FAIR tooling (54), and FAIR service support (55, 56), here we restrict to reporting the few successful experiences that have build FAIR-compliant infrastructures in very specific and practical scenarios.

A preliminary effort of nine Dutch labs aimed to publicly share variant classifications (even if at the time the “FAIR” concept had not been developed yet) (57); the work was expanded 2 years later in the context of the “Rational Pharmacotherapy Program” (The Netherlands Organization for Health Research and Development), developing an instruction manual for FAIR genomic data in clinical care and research—this was based on an inventory of commonly used workflows and standards in the broader genome analysis (58). The same authors finally proposed a FAIR Genomes metadata schema, specifically focusing on promoting genomic data reuse in the Dutch healthcare ecosystem (59). Parallel efforts were devoted to analysis in distributed platforms for radiomics (60) and leukodystrophy (61). ETL processes that are compliant with FHIR were proposed in Peng et al. (62) and Van Damme et al. (63).

To the best of our knowledge, a large, coordinated European-level effort of FAIRification, such as the one proposed in BETTER –with the goal of enabling better distributed analytics—has not been achieved yet in a documented way. BETTER aims to describe all data in a standardized comprehensive manner, so as to process the data in the trains with the most current machine learning available models. Integral analyzes performed on secure systems will provide insight into disease for large cohorts of patients, with significant impact.

### 4.2 Data fusion

To gain the maximum from data, an important step is data fusion. Data fusion is the process of integrating multiple data sources to produce more consistent, accurate, and useful information than that provided by any single data source. Local data fusion consists of integrating data from multiple sources within a single institution (or Station). This type of data fusion is useful when the data sources are heterogeneous, such as genomic, clinical, and phenotypic data of the same patient. Using AI-based solutions to integrate data heterogeneous data enables the creation of complementary, cohesive, and more complete information, which leads to more accurate insights (64). Moreover, we plan to develop frameworks and methods to also allow us to integrate patients' data from wearable devices and smartphones. Distributed data fusion is the task of integrating data from multiple institutions (Stations). While local data fusion is well-established, distributed fusion is a fairly novel discipline and contains large potentials (65, 66). We envision two main applications for distributed data fusion: integration of homogeneous or heterogeneous data sources. The first aims at creating larger cohorts by combining data provided by independent institutions and removing potential biases due to different collection protocols or techniques. Examples are batch removal algorithms for genomic data (12) or image registration (67). Several methods based on AI exist to perform such tasks and are commonly used by the research community; the challenges for the BETTER architecture consist in designing and developing approaches to achieve the same results in a distributed context, where no data sharing is allowed. The second application can be used to combine several data modalities, each providing different viewpoints on a common phenomenon to solve inference and knowledge discovery tasks. The ambition is to fuse several dimensions including laboratory analysis, medical reports, drug therapy, imaging, genomics, socio-demographic, geographical, and medical questionnaires. To this aim, we also plan to investigate the availability and fuse publicly accessible data sources. In the context of the project, we will develop and implement AI-based solutions tailored to the clinical use cases, e.g., intended to perform analyzes on clusters of interest or compare different therapeutic regimens. Finally, we plan to adopt AI to generate several synthetic datasets, using generative AI and data augmentation approaches to be released to the community for developing medical AI-based solutions.

## 5 Conclusion

The BETTER project relies on the concept of "bringing computation to data" through incremental and federated learning. This approach avoids unnecessary data transfers between medical centers while effectively utilizing the encoded information within. The project builds upon the experience gained from previous initiatives like the PADME and Vantage6 projects, as well as from the health/genomic data integration expertise of the Data-driven Genomic Computing project.

Aligned with the European Health Data Space (EHDS), BETTER aims to empower EU medical centers and beyond to fully exploit the potential of securely exchanging, utilizing, and reusing health data, facilitated by robust data FAIRification.

Starting from the domains of intellectual disability, inherited retinal dystrophies, and autism spectrum disorders—with potential expansion to other diseases —the analytical tools developed will enhance healthcare professionals' proficiency in cutting-edge digital technologies, data-driven decision support, health risk surveillance, and healthcare quality monitoring and management. These advancements are expected to positively impact health policymakers and innovators alike.

## Data Availability

The original contributions presented in the study are included in the article/supplementary material, further inquiries can be directed to the corresponding author.
